# A Mathematical Theory of Cortex-Receptor Artificial Extension

**DOI:** 10.1038/s41598-020-57591-w

**Published:** 2020-01-21

**Authors:** You-Lu Xing

**Affiliations:** Department of Computer Science and Technology, Nanjing University, Nanjing, 210023 China

**Keywords:** Computational science, Computer science

## Abstract

Many physiology experiments demonstrate that an organism’s cortex and receptor system can be artificially extended, giving the organism new types of perceptual capabilities. To examine artificial extension of the cortex-receptor system, I propose a computational model that allows new types of sensory pathways to be added directly to the computational model itself in an online manner. A synapse expandable artificial neuron model that can grow new synapses, forming a bridge between the novel perceptual information and the existing neural network is introduced to absorb the novel sensory pathway. The experimental results show that the computational model can effectively integrate sudden emerged sensory channels and the neural circuits in the computational model can be reused for novel modalities without influencing the original modality.

## Introduction

Many physiological experiments have demonstrated the expandability of an organism’s cortex-receptor system. Human L-pigment gene knock-in mice, which express a human long-wavelength-sensitive cone photopigment, acquired a new capacity for chromatic discrimination^1^. Rats that receive information from the infrared environment in their somatosensory cortex can perceive infrared light^2^. Monkeys have learned to discriminate artificial tactile stimuli in an active tactile exploration task, where a brain-machine-brain interface was used to deliver the stimuli to their primary somatosensory cortex^3^. The rat and monkey experiments imply that the sensing capability of an organism can be expanded artificially throughout the organism’s lifetime, differing from the natural way, where organisms acquire new experiences through genetic variation and those sensory transducers are fixed at birth^4,5^. To provide a mathematical theory for the cortex-receptor artificial extension, in this study, I build a computational model that can adapt to a novel sensory pathway in an online manner.

**Figure 1:**
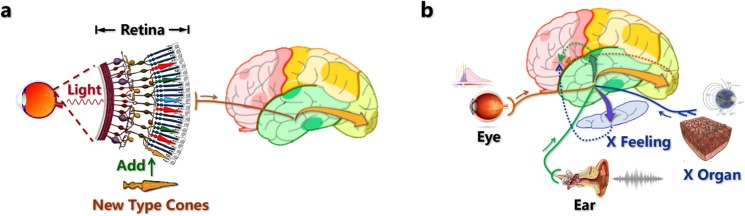
Two situations of extensions of the cortex-receptor system. (**a**) Emergence of novel sensory receptors in an existing perceptual channel. The figure illustrates a new type of cone cells being added to the retina, which is inspired by the experiment in which human L-pigment gene knock-in mice showed enhanced long wavelength sensitivity^1^. In this situation, novel receptors receive environmental stimuli and transmit signals synchronously with the pre-existing receptors, which means that the perceptual channel spectrum is expanded. (**b**) Emergence of novel perceptual channels in the whole system. The figure illustrates a new type of sensory channel X being added to the organism and the channel communicates with other parts of the cortex that are responsible for different sensory types. As a result, the organism gains the ability to sense X. This situation corresponds to the experiment in which rats were able to perceive infrared light through electrodes implanted in their cortex^2^ and monkeys were able to feel artificial tactile stimuli via a brain-machine-brain interface^3^.

According to the experiments^1–3^, I divide the extension into two situations: (i) emergence of novel sensory receptors in an existing perceptual channel (Fig. 1a) and (ii) emergence of novel perceptual channels in the whole system (Fig. 1b).

Inspired by the phenomenon that novel experience can induce a formation of new spines in the brain^6,7^, a synapse expandable artificial neuron model that can grow new synapses to absorb extended sensory signal during learning is designed. The model is different from the classic artificial neuron model whose synapse structure is fixed^8^, which implies a non-extendibility for the novel sensory signals. Meanwhile, to enable the extension of the sensory pathway in the above two situations, a hierarchical and modularized computational structure inspired by the brain structure^9^ is designed. The synapse expandable artificial neuron can work on any level of the computational structure, which provides an adequate theoretical explanation to the cortex-receptor artificial extension.

## Results

### Computational framework

**Figure 2:**
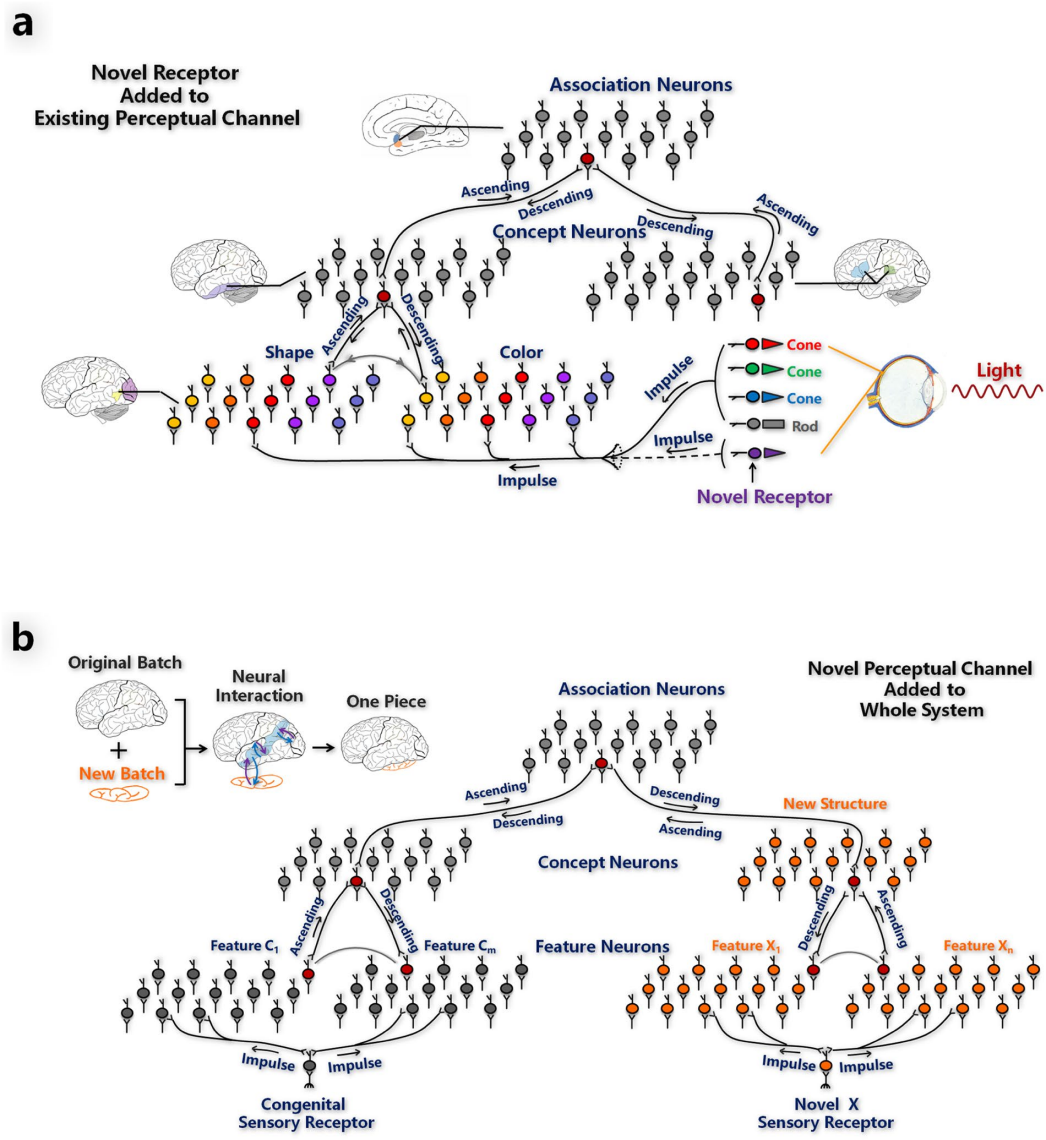
Computational framework of extensions to the cortex-receptor system. (**a**) A novel sensory receptor is added to an existing perceptual channel. (**b**) A novel perceptual channel is added to the entire system.

The computational framework for situation (i) (Fig. 2a) and situation (ii) (Fig. 2b) is based on a perception coordination network^10^, which is a hierarchical and modularized neural network (see Methods section perception coordination network for algorithmic details). The framework includes the primary sensory areas, the unimodal association areas, and the multimodal association areas. The primary sensory areas contain feature neurons, that respond to elementary features, for example, color, shape, or syllable features. The unimodal association areas contain concept neurons, which combine the elementary features to represent unimodal object concepts; for example, in the visual channel, they combine information from color and shape neurons to represent object images. The multimodal association areas contain association neurons, which connect concept neurons in different perceptual channels, for example, connecting concept neurons in visual and auditory channels to associate the visual and auditory concepts of objects. Figure 2a shows an example of a novel type of receptor added to the retina of an organism, which receives stimuli synchronously with the inherent receptors. Impulses generated by both the novel and inherent receptors are transmitted to the primary sensory areas where they are integrated. Figure 2b shows a novel perceptual channel X being added to the network, which then receives stimuli synchronously with the congenital perceptual channels. Impulses generated by both the novel and congenital channels are transmitted to the primary sensory areas and unimodal association areas, respectively, and are finally combined in the multimodal association area. The neural interactions launched by the association neurons, working as an adhesive, integrates new structure into the original structure to form a single system.

### Synapse expandable artificial neuron model

**Figure 3:**
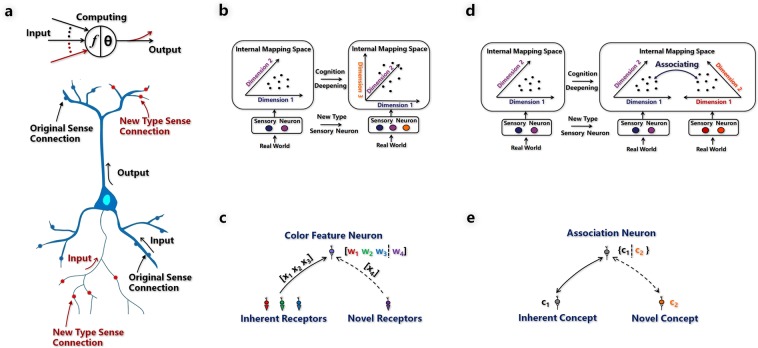
Computational solutions. (**a**) Synapse-expandable artificial neuron model. New synapses (shown in red) can be created to allow a pathway for novel perceptual receptors or channels. (**b**) Mathematical modelling of situation (i). After a new type of sensory receptor is added to an existing perceptual channel, the organism has a new way to perceive the environment. Consequently, the internal mapping space of the organism has a corresponding new dimension. Thus, the points that represent the learned concepts in the original low dimension feature space are mapped to a higher-dimensional feature space. (**c**) The dimensionality increasing process of the feature neuron. Feature neuron grows new synapses to absorb novel features. (**d**) Mathematical modelling of situation (ii). After a new perceptual channel is added to the entire system, the internal mapping space of the organism has a corresponding new space, and new concepts will be generated in this space. Finally, the concepts in the original space and the new space become associated with each other. (**e**) The response modal expansion process of the association neuron. Association neuron grows new synapses to absorb novel modality concept.

To integrate novel sensory receptors or perceptual channels into the pre-existing neural network, I design a synapse expandable artificial neuron model (Fig. 3a). New synapses (shown in red) can be created to allow pathways for the novel receptor and novel perceptual channel. The expanded synapses on the dendrite (input) side can transmit signals received from new types of sensory receptors. Then, the new type sense has a signal pathway to the existing system; the expanded synapses on the axon (output) side can transmit signals from the existing system to the new type of sense channel. Then, the system has a signal pathway to the new type sense channel. When new synapses are created, the activation function of the synapse expandable artificial neuron model evolves as follows,

1 $${{\boldsymbol{z}}}_{0}={\boldsymbol{f}}({{\boldsymbol{x}}}_{0},{{\boldsymbol{w}}}_{0},{\boldsymbol{\theta }})\to [{{\boldsymbol{z}}}_{0},{{\boldsymbol{z}}}_{1}]={\boldsymbol{f}}([{{\boldsymbol{x}}}_{0},{{\boldsymbol{x}}}_{1}],[{{\boldsymbol{w}}}_{0},{{\boldsymbol{w}}}_{1}],{\boldsymbol{\theta }})$$

where ${{\boldsymbol{x}}}_{0}$ and ${{\boldsymbol{z}}}_{{\bf{0}}}$ are the input and output in the original synapses, respectively, ${{\boldsymbol{w}}}_{0}$ is the weights of the original input synapses, ${{\boldsymbol{x}}}_{{\bf{1}}}$ and ${{\boldsymbol{z}}}_{{\bf{1}}}$ are the input and output in the new synapses, respectively, ${{\boldsymbol{w}}}_{{\bf{1}}}$ is the weights of the new input synapses, *θ* is the threshold of the artificial neuron. The implanted electrodes in the IR rat experiment^2^ can be regarded as another form of synapses. Then, a special case of the activation function can be used to explain the IR rat system, where ${{\boldsymbol{x}}}_{{\bf{1}}}$ equals the input infrared light, ${{\boldsymbol{w}}}_{{\bf{1}}}$ transforms the input infrared signal ${{\boldsymbol{x}}}_{{\bf{1}}}$ to the microstimulation which is sent to the neuron, and ${{\boldsymbol{z}}}_{{\bf{1}}}$ is empty here because the IR rat system reuses the original output pathway of the neuron with high probability.

### Novel receptor added to existing perceptual channel

Assume that a perceptual channel initially has *n* sensory receptors, that receive an *n*- dimensional vector ${{\boldsymbol{x}}}_{{\bf{I}}}=({x}_{1},{x}_{2},\ldots ,{x}_{n})\in {{\bf{R}}}^{n}$. After $m$ new sensory receptors, which receives an *m*-dimensional vector ${{\boldsymbol{x}}}_{{\bf{II}}}=({x}_{n+1},{x}_{n+2},\ldots ,{x}_{n+m})\in {{\bf{R}}}^{m}$, are added to the perceptual channel, the dimension of the internal mapping space of the channel grows from ${{\bf{R}}}^{n}$ to ${{\bf{R}}}^{n+m}$, and the learned concepts in space ${{\bf{R}}}^{n}$ are mapped to a higher-dimensional feature space ${{\bf{R}}}^{n+m}$, as illustrated in Fig. 3b. For example, assuming that novel receptors that receive very short wavelength light are added to a trichromatic color vision organism, the organism’s color perception will evolve from the original 3-dimensional system (R, G, B) to a 4-dimensional system (R, G, B, X). Feature neurons are used to realize the mapping from space ${{\bf{R}}}^{n}$ to space ${{\bf{R}}}^{n+m}$, and new synapses are created between the new sensory neurons and the feature neurons for the ${{\boldsymbol{x}}}_{{\bf{II}}}$ pathway, as illustrated in Fig. 3c. The main idea of the algorithm is as follows. When an input sample $({{\boldsymbol{x}}}_{{\bf{I}}},{{\boldsymbol{x}}}_{{\bf{II}}})$ arrives, feature extraction is conducted using ${{\boldsymbol{x}}}_{{\bf{I}}}$ and ${{\boldsymbol{x}}}_{{\bf{II}}}$. I assume that the feature vectors ${{\boldsymbol{y}}}_{{\bf{I}}}$ and ${{\boldsymbol{y}}}_{{\bf{II}}}$ are obtained through ${{\boldsymbol{x}}}_{{\bf{I}}}$ and ${{\boldsymbol{x}}}_{{\bf{II}}}$, respectively. Then, ${{\boldsymbol{y}}}_{{\bf{I}}}$ tries to activate a feature neuron in its corresponding primary sensory areas. When some feature neuron is activated but no synapse exists between the feature neuron and the novel sensory neurons, new synapses are created between them and the weights of the synapses are initialized to ${{\boldsymbol{y}}}_{{\bf{II}}}$, which means that ${\boldsymbol{w}}=({{\boldsymbol{w}}}_{{\bf{I}}},{{\boldsymbol{w}}}_{{\bf{I}}{\bf{I}}})$, where ${{\boldsymbol{w}}}_{{\bf{I}}}$ represents the original weights of the feature neuron, ${{\boldsymbol{w}}}_{{\bf{I}}{\bf{I}}}$ represents the original weights of the feature neuron which is initialized to ${{\boldsymbol{y}}}_{{\bf{II}}}$. According to Eq. (1), the activation function of the feature neuron evolves as follows,

2 $${\boldsymbol{z}}={\boldsymbol{f}}({{\boldsymbol{x}}}_{{\bf{I}}},{{\boldsymbol{w}}}_{{\bf{I}}},{\boldsymbol{\theta }})\to {\boldsymbol{z}}=f([{{\boldsymbol{x}}}_{{\bf{I}}},{{\boldsymbol{x}}}_{{\bf{I}}{\bf{I}}}],[{{\boldsymbol{w}}}_{{\bf{I}}},{{\boldsymbol{y}}}_{{\bf{I}}{\bf{I}}}],{\boldsymbol{\theta }})$$

where ${{\boldsymbol{x}}}_{{\bf{I}}}$ and ${{\boldsymbol{w}}}_{{\bf{I}}}$ are the input and weights in the original synapses, respectively. ${{\boldsymbol{x}}}_{{\bf{I}}{\bf{I}}}$ and ${{\boldsymbol{y}}}_{{\bf{I}}{\bf{I}}}$ are the input and initialized weights in the new synapses, respectively, *θ* is the threshold of the artificial neuron. ***z*** is the output of the neuron. Finally, a deeper feature reflecting the external world is formed in the network, and the feature neuron can perform calculations in the high dimension feature space (see Methods section dimensionality increasing process of the feature neurons for algorithmic details).

### Novel perceptual channel added to whole system

Assume that the system has an inherent perceptual channel ${\bf{L}}$, that receives an *n*-dimensional vector ${{\boldsymbol{x}}}^{{\bf{I}}}=({x}_{1}^{{\bf{I}}},{x}_{2}^{{\bf{I}}},\ldots ,{x}_{n}^{{\bf{I}}})\in {{\bf{R}}}^{n}$. After a new perceptual channel **X**_,_ which receives an *m*-dimensional vector ${{\boldsymbol{x}}}^{{\bf{II}}}=({x}_{1}^{{\bf{II}}},{x}_{2}^{{\bf{II}}},\ldots ,{x}_{m}^{{\bf{II}}})\in {{\bf{R}}}^{m}$, is added to the system, a new internal mapping space emerges in the system. The system should associate concepts in the new space with the concepts in the inherent space, as illustrated in Fig. 3d. Association neurons are used to associate the concepts in the two spaces by creating synapses between the concept neurons in channel **X** and the association neurons that already connect to some concept neurons in the inherent perceptual channel, as illustrated in Fig. 3e. This process is based on the Hebbian theory that neurons that fire together wire together. The main idea of the algorithm are as follows. When an input sample pair $({{\boldsymbol{x}}}^{{\bf{I}}},{{\boldsymbol{x}}}^{{\bf{II}}})$ arrives, ${{\boldsymbol{x}}}^{{\bf{I}}}$ tries to activate some association neuron through the inherent perceptual channel. If the activated association neuron is not connected with any concept neurons in channel ${\bf{X}}$, a new synapse is created between the association neuron and the concept neuron, which is activated by ${{\boldsymbol{x}}}^{{\bf{II}}}$ in channel ${\bf{X}}$. According to Eq. (1), the activation function of the association neuron evolves as follows,

3 $${{\boldsymbol{z}}}^{{\bf{I}}}={\boldsymbol{f}}({{\boldsymbol{x}}}^{{\bf{I}}},{{\boldsymbol{w}}}^{{\bf{I}}},{\boldsymbol{\theta }})\to [{{\boldsymbol{z}}}^{{\bf{I}}},{{\boldsymbol{z}}}^{{\bf{I}}{\bf{I}}}]={\boldsymbol{f}}([\,{{\boldsymbol{x}}}^{{\bf{I}}},{{\boldsymbol{x}}}^{{\bf{I}}{\bf{I}}}],[{{\boldsymbol{w}}}^{{\bf{I}}},{{\boldsymbol{w}}}^{{\bf{I}}{\bf{I}}}],{\boldsymbol{\theta }})$$

where ${{\boldsymbol{z}}}^{{\bf{I}}{\bf{I}}}$ is the new output synapse to channel ${\bf{X}}$ which provides a signal pathway from channel ${\bf{L}}$ to channel ${\bf{X}}$. Meanwhile, ${{\boldsymbol{z}}}^{{\bf{I}}}$ can provide a reverse signal pathway from channel ${\bf{X}}$ to channel ${\bf{L}}$ now. ***x***^**I**^ and ***x***^**II**^ are the input from the concept neurons in ${\bf{L}}$ and ${\bf{X}}$, respectively. ${{\boldsymbol{w}}}^{{\bf{I}}}$ and ${{\boldsymbol{w}}}^{{\bf{I}}{\bf{I}}}$ are the weights of the synapses from the concept neurons in ${\bf{L}}$ and ${\bf{X}}$ to the association neuron, respectively. These synapses are two independent groups. Subsequently, the system can respond to the new perceptual channel, which means that the system can respond to a new world by feeling ${\bf{X}}$ (see Materials and Methods section response modal expansion of the association neurons for algorithmic details).

### Experiments

**Figure 4:**
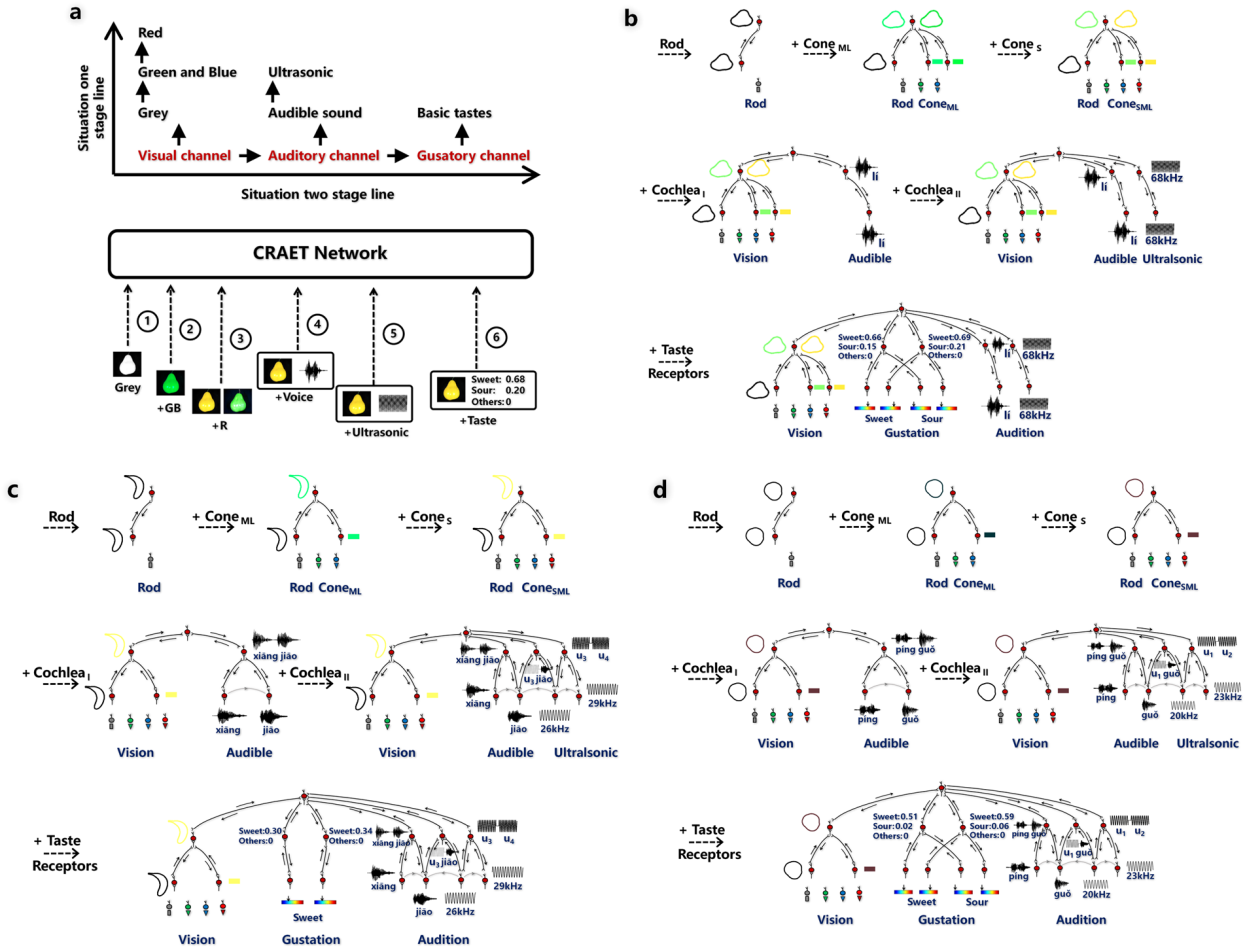
Experiment. (**a**) Experimental settings. (**b**–**d**) Neurons in the learned network that associate with concept pear, banana and apple. The icons next to the neuron are the objects to which the neuron maximally responds. Thresholds for neurons to be activated are set as follows: in the visual channel, thresholds of the shape and color neurons are 1/4 times the L2 norm of their weights; in the auditory channel, thresholds of the audible and ultrasonic syllable neurons are 200 and 9, respectively; in the gustatory channel, thresholds of the basic flavor neurons are 0.015.

An experiment (Supplementary Fig. 1 shows the objects used) is designed to validate our computational model, which is called the CRAET (cortex-receptor artificial extension theory) network. Figure 4a shows the experimental setting, I first give the computational model a visual channel. To simulate the visual system evolving from achromatopsia to dichromatopsia and then to trichromatopsia, I initially give the visual channel a brightness receptor (corresponding to the Rods) that receives grayscale object images, and let the network learn object shapes. After a period of learning with all objects, I provide the visual channel with new green and blue color receptors (corresponding to the M-Cones and L-Cones) so that it additionally receives the green and blue light, respectively. Now, the network can receive a grey + green + blue image of the object. Then, I let the network perceive all objects again to learn color information of each object. After that, I add a red color receptor (corresponding to the S-Cones), allowing it to receive the red light. The network can now receive a Grey + RGB image of the object. Similarly, I let the network perceive all objects another round to update its color feature neurons. Next, I introduce an auditory channel that enables audible sound input to the network. At this time, visual and auditory input can be received simultaneously. I let the network learn the Chinese name of each object by receiving pairs consisting of images accompanied by the Chinese names of each object. After all objects are learned, I add an ultrasonic receptor to the auditory channel and use ultrasonic sounds to name all objects again. Finally, a gustatory channel is added to the network. Pairs of image and taste samples are provided to the network simultaneously at this stage to let the network learn the taste of each object.

Figure 4b shows the neurons in the learned network that associate with concept pear. The top line shows the change of the network structure of the concept pear as the network acquires new visual receptors. Initially, the network receives only a greyscale image of the object and learns the shape of the object. After the green and blue color receptors are added, the network can receive certain object colors, and color feature neurons are created in the network. The visual concept neuron is then able to respond to color information. In this case, two colors are associated with the pear shape, one appears a little brighter and the other is somewhat darker. However, they are not easy to distinguish in this color space. After the red color receptor is added, the color feature neurons are mapped to a higher-dimensional space: the RGB color space. One color feature neuron becomes responsive to yellow, and another becomes responsive to green. Thus, the bright green pear and dark green pear become more easily distinguishable in the RGB color space: one is yellow, and the other is green. This gives trichromatic organisms an advantage compared to dichromatic organisms. The middle line shows the change in the network structure of the concept pear after adding an auditory channel. On the left, the auditory channel which provides audible frequencies is added. On the right, the auditory receptor which provides ultrasonic frequencies is added. The association neuron connects the images and names (audible and artificial ultrasonic words) of the object correctly. The bottom line shows the change in the network structure of the concept pear after a gustatory channel is added. The artificial taste data of the pear contain sweet and sour flavors. The network learns these two flavors (sweet 0.67, sour 0.22) and (sweet 0.63, sour 0.14). The response modal of the associate neuron is expanded with the concept of taste. Figure 4c,d show two similar results. The experiment demonstrates the CRAET network can effectively integrate newly introduced sensory receptors and channels in an online manner (Supplementary Fig. 2 shows more learning results).

**Figure 5:**
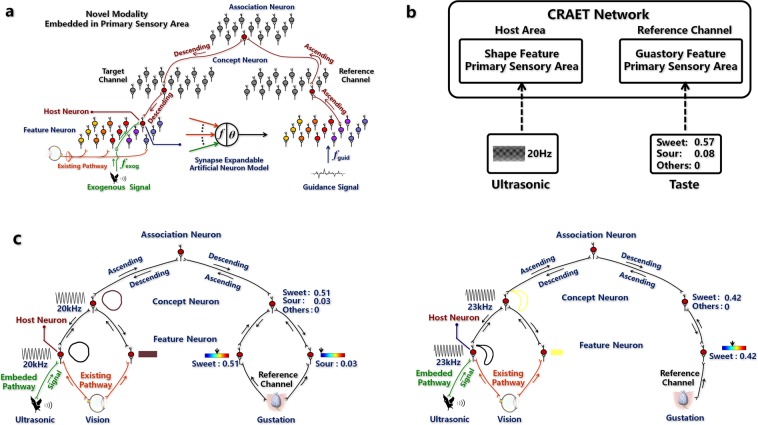
Modality embedding experiment. (**a**) Computational framework of the modality embedding. (**b**) Experimental settings. I set the gustatory channel as the reference channel and the visual channel as the target channel. The ultrasonic modality is embedded into the shape feature primary sensory area. (**c**) Examples of the embedding result. The shape feature neurons are embedded with an ultrasonic modality without being deprived of their original receptive field, which means the ultrasonic modality reuses the vision-gustation circuit effectively. Thresholds for neurons to be activated are set as follows: thresholds of the ultrasonic syllable neurons are 200 and 9, respectively; thresholds of the basic flavor neurons are 0.015.

Inspired by the phenomenon found in the IR rat experiment in which a new receptive field was embedded in S1 neurons without hijacking their original receptive field^2^, I designed a modality embedding experiment. Figure 5a shows the computational modelling. The network receives a pair of samples that includes an exogenous signal ${{\boldsymbol{x}}}_{{\boldsymbol{e}}}$ to be embedded and a guidance signal ${{\boldsymbol{x}}}_{{\boldsymbol{g}}}$, which leads ${{\boldsymbol{x}}}_{{\boldsymbol{e}}}$ to the host neuron. The guidance signal first transmits in ascending fashion in the reference channel to activate some association neuron. Then, the activated association neuron transmits signals in descending fashion to the host area, where the new modality is embedded, to activate some host neuron used to absorb the novel modality. If the host neuron does not have synapses connected to the novel modal signal pathway, new synapses will be created, and their weights will be initialized as ${\boldsymbol{w}}={f}_{exog}({{\boldsymbol{x}}}_{{\boldsymbol{e}}})$ to store the pattern of the exogenous signal ${{\boldsymbol{x}}}_{{\boldsymbol{e}}}$, where ${f}_{exog}$(·) is the feature extraction function of the novel modality. If the host neuron already has synapses connected to the novel modal signal pathway, the weights of the synapses are updated using the competitive learning rule, ${\boldsymbol{w}}={\boldsymbol{w}}+\delta ({f}_{exog}({{\boldsymbol{x}}}_{{\boldsymbol{e}}})-{\boldsymbol{w}})$, where *δ* is the learning rate (see Methods section modality embedding for algorithmic details).

Figure 5b shows the experimental setting, where an ultrasonic modality is embedded into the neurons in the shape feature primary sensory area using a taste sample as a guidance signal. Figure 5c shows two examples of embedding results. In the results, the shape feature neurons are embedded with an ultrasonic modality without being deprived of their original receptive field. The ultrasonic modality reuses the vision-gustation circuit effectively (Supplementary Fig. 3 shows more embedding results).

## Discussion

In this study, a mathematical theory for the cortex-receptor artificial extension is studied. I design a synapse expandable artificial neuron mode to absorb novel information flow. A hierarchical and modularized computational structure is proposed to enable novel information flow be integrated in different concept levels. Meanwhile, I design different computational models for different types of neurons found in many physiological experiments including the feature neurons^11^, concept neurons^12^ and association neurons^13^ (see Methods section perception coordination network for different neuron models).

Currently, the computational mechanism is built only at the neuron level. The computational mechanism of the cortex and receptor extension at the large-scale neural circuit level should be studied. This raises an interesting question: can we design new modality feelings with electronic circuits and then integrate them into organisms? Regarding the question of whether organisms experience novel sensory inputs as a new distal sensory modality^2^, I feel the computational and behavioral dimensions of this question are extremely interesting avenues for future research, with particular potential to be studied in human subjects. Organisms’ cortex and receptor systems tend to become more complex over time; As more information arrives at the brains of the organisms, and in response, there is increasing number of information processing modes of the brain which seem to become increasingly complicated. The organisms are able to understand the real world more deeply. The brain-machine interface experiments^2,3^ showed the potential to expand a species’ normal perceptual range. Building up a corresponding computational theory will deepen our understanding about the extensibility of the central nervous system and the organism-machine hybrid intelligence. It seems clear from experimental results that we have not come close to exhausting the potential for incorporating novel sources of information into cortical processing modes^14^, this also raises a very interesting question: can we know or prove that we can already perceive all the dimensions of the world?

## Methods

### Perception coordination network

**Figure 6:**
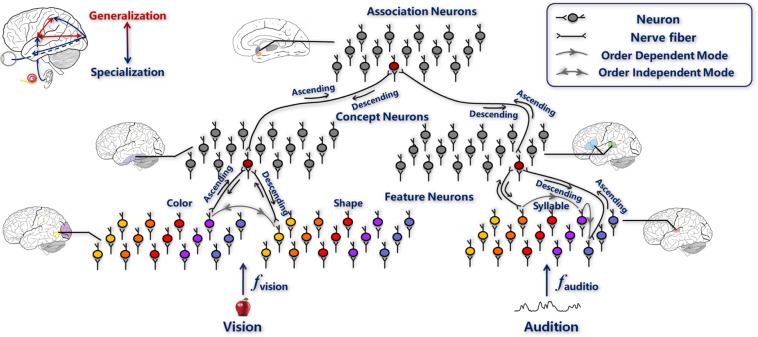
Computational model of the perception coordination network. The hierarchical and modularized structure of the network is inspired by the brain’s structure. Note that the figure depicts only vision and audition, other sensations could also be included in the structure.

The perception coordination network is an online learning framework that aims to incrementally learn and bind concepts. As shown in Fig. 6, the network includes the primary sensory areas, the unimodal association areas and the multimodal association areas.

The primary sensory areas include feature neurons that respond to particular features, for example, shape features, color features, or syllable features. Feature neurons that respond to the same feature type are located in the same area *α*, and I use the set ${N}^{{F}_{\alpha }}$ to represent them. As mentioned above, *α* can be the shape area ${b}$, the color area ${c}$, or the syllable area ${s}$. ${N}_{i}^{{F}_{\alpha }}$ denotes feature neuron ${i}$ in area *α* and ${N}_{i}^{{F}_{\alpha }}=\{{{\boldsymbol{w}}}_{{i}},{\sigma }_{i}\}$, where ${{\boldsymbol{w}}}_{{i}}$ represents the weights and ${\sigma }_{i}$ is the cumulative number of times the neuron has been activated. The activation function of ${N}_{i}^{{F}_{\alpha }}$ is $$f({\boldsymbol{x}})=\{\begin{array}{ll}1, & \parallel {\boldsymbol{x}}-{{\boldsymbol{w}}}_{i}\parallel \le \theta \\ 0, & \parallel {\boldsymbol{x}}-{{\boldsymbol{w}}}_{i}\parallel  > \theta \end{array}$$, where $\theta$ is a parameter that controls the response range of the feature neuron.

The unimodal association areas include concept neurons, which connect the feature neurons to represent a unimodal concept, for example, to form visual concepts by connecting shape and color feature neurons, or to form words (auditory concepts) by connecting syllable feature neurons. Concept neurons with the same modality are located in the same area, $\beta$, and I use set ${N}^{{C}_{\beta }}$ to represent them. $\beta$ can be a visual association area ${v}$, an auditory association area ${a}$, or some other sensory association area. ${N}_{i}^{{C}_{\beta }}$ denotes the concept neuron ${i}$ in area $\beta$. The activation mode of the concept neuron is one of two types: order-independent activation mode and order-dependent activation mode. For example, a visual concept neuron has an order-independent activation mode because different sequences in the activation of shape and color feature neurons to which a visual concept neuron connects do not affect the activation of the visual concept neuron. In contrast, auditory concept neurons (for example, words) have an order-dependent activation mode because a word is composed of a sequence of syllables. Correspondingly, two types of circuit structure between a concept neuron and a feature neuron are defined as followswhere the arrows represent connections between neurons. Then, the activation function of ${N}_{i}^{{C}_{\beta }}$ is $$f({N}_{1}^{{F}_{\alpha }},{N}_{2}^{{F}_{\alpha }},\ldots ,{N}_{n}^{{F}_{\alpha }})=\{\begin{array}{ll}1, & {N}_{i}^{{F}_{\alpha }}=1,1\le i\le n\\ 0, & {\rm{else}}\end{array}$$, where feature neurons ${{N}}_{1}^{{{F}}_{{\alpha }}}$, ${{N}}_{2}^{{{F}}_{{\alpha }}}$, …, ${{N}}_{{n}}^{{{F}}_{{\alpha }}}$ connect to ${N}_{i}^{{C}_{\beta }}$, and ${{N}}_{{i}}^{{{F}}_{{\alpha }}}=1$ means ${{N}}_{{i}}^{{{F}}_{{\alpha }}}$ is activated. For the order-dependent activation mode, ${{N}}_{1}^{{{F}}_{{\alpha }}}$, ${{N}}_{2}^{{{F}}_{{\alpha }}}$, …, ${{N}}_{{n}}^{{{F}}_{{\alpha }}}$ must be activated in sequence. The connection between concept neuron ${N}_{i}^{{C}_{\beta }}$ and feature neuron ${{N}}_{{j}}^{{{F}}_{{\alpha }}}$ is denoted as ${c}_{(i,j)}=\{{{N}}_{{i}}^{{{C}}_{\beta }},{{N}}_{{j}}^{{{F}}_{{\alpha }}},{\rho }_{(i,j)}\}$, where ${\rho }_{(i,j)}$ is the cumulative number of times the connection is activated.

The multimodal association areas include association neurons, which connect the concept neurons in different unimodal association areas, for example, they connect an image of an object represented by a visual concept neuron with its name, which is represented by an auditory concept neuron. The association neurons are stored in set ${N}^{A}$, and ${N}_{i}^{A}$ is used to denote the association neuron for ${i}$. There are four types of circuit structures between an association neuron and a concept neuron,Each concept neuron in the circuit can activate the association neuron. Thus, the association neuron has a multimodality activation mode, and the activation function of ${N}_{i}^{A}$ is $$f({{N}}_{1}^{{{C}}_{\beta }},{{N}}_{2}^{{{C}}_{\beta }},\ldots ,{{N}}_{{n}}^{{{C}}_{\beta }})=\{\begin{array}{ll}1, & {{N}}_{{i}}^{{{C}}_{\beta }}=1,i\in \{1,2,\ldots ,{n}\}\\ 0, & else\end{array}$$, where the concept neurons ${{N}}_{1}^{{{C}}_{\beta }}$, ${{N}}_{2}^{{{C}}_{\beta }}$, …, ${{N}}_{{n}}^{{{C}}_{\beta }}$ connect to ${N}_{i}^{A}$, and ${{N}}_{{i}}^{{{C}}_{\beta }}=1$ means ${{N}}_{{i}}^{{{C}}_{\beta }}$ is activated. The connection between concept neuron ${N}_{m}^{{C}_{\beta }}$ and another concept neuron ${N}_{n}^{{C}_{\beta }}$ through association neuron ${N}_{i}^{A}$ is denoted as ${c}_{(m,i,n)}=\{{{N}}_{{m}}^{{{C}}_{\beta }},{{N}}_{{i}}^{{A}},{{N}}_{{n}}^{{{C}}_{\beta }},{\rho }_{(m,i,n)}\}$, where ${\rho }_{(m,i,n)}$ is the cumulative number of times the connection is activated.

Without loss of generality, in the following I use a pair of visual (an object image) and auditory (the name of an object) inputs to describe the method in detail.

When an image and voice (name) pair arrives, feature extraction is conducted first. Currently, the normalized Fourier descriptors of the object’s boundary ***d*** and the color histogram of the object ***h*** are used for visual features. The Mel-Frequency Cepstral Coefficients (MFCCs) of the syllables contained in the voice ***m*** are used for the auditory features, where short-time energy and short-time zero crossing are used to extract the syllables from a voice wave. Then, a competitive learning process is executed among the feature neurons.

In the visual channel, a winner neuron ${N}_{b}^{{F}_{b}}$ in the shape feature area and a winner neuron ${N}_{c}^{{F}_{c}}$ in the color feature area are found using ${{N}}_{b}^{{{F}}_{b}}=\mathop{{\rm{\arg }}\,{\rm{\min }}}\limits_{{{N}}_{{i}}^{{{F}}_{b}}\in {{N}}^{{{F}}_{b}}}\,\parallel {\boldsymbol{d}}-{{\boldsymbol{w}}}_{i}{\parallel }_{2}$ and ${{N}}_{c}^{{{F}}_{c}}=\mathop{{\rm{\arg }}\,{\rm{\min }}}\limits_{{{N}}_{{i}}^{{{F}}_{c}}\in {{N}}^{{{F}}_{c}}}\,\parallel {\boldsymbol{h}}-{{\boldsymbol{w}}}_{i}{\parallel }_{2}$, where ${N}^{{F}_{b}}$ and ${N}^{{F}_{c}}$ are the sets of neurons in the shape area and color area, respectively. In the shape area, if $\parallel {\boldsymbol{d}}-{{\boldsymbol{w}}}_{b}{\parallel }_{2}\le \frac{1}{4}\parallel {{\boldsymbol{w}}}_{b}{\parallel }_{2}$, ${N}_{b}^{{F}_{b}}$ is activated and updated using the winner-take-all principle: ${\sigma }_{b}={\sigma }_{b}+1$, ${{\boldsymbol{w}}}_{b}={{\boldsymbol{w}}}_{b}+({\boldsymbol{d}}-{{\boldsymbol{w}}}_{b})/{\sigma }_{b}$; If $\parallel {\boldsymbol{d}}-{{\boldsymbol{w}}}_{b}{\parallel }_{2} > \frac{1}{4}\parallel {{\boldsymbol{w}}}_{b}{\parallel }_{2}$, the network recognizes ***d*** as a new feature and creates a new feature neuron to record it: ${N}_{new}^{{F}_{b}}=\{{\boldsymbol{h}},1\}$. Then, ${N}_{new}^{{F}_{b}}$ is activated. A similar process is executed in the color area. Finally, the activated feature neurons in the shape area (assumed to be ${N}_{{{f}}_{{b}}}^{{F}_{b}}$) and color area (assumed to be ${N}_{{{f}}_{{c}}}^{{F}_{c}}$) transmit their activation signals to the visual unimodal association area.

In the auditory channel, dynamic time warping (DTW) is used to find the winner neuron for each syllable contained in the voice input: ${{N}}_{{f}_{i}}^{{{F}}_{s}}=\mathop{{\rm{\arg }}\,{\rm{\min }}}\limits_{{{N}}_{{j}}^{{{F}}_{s}}\in {{N}}^{{{F}}_{s}}}\,{\bf{d}}{\bf{t}}{\bf{w}}({{\boldsymbol{m}}}_{i},{{\boldsymbol{w}}}_{j})$, where $1\le i\le k$, ${{\boldsymbol{w}}}_{j}$ represents the MFCCs of syllable feature neuron ${j}$, ${{\boldsymbol{m}}}_{i}$ is the MFCCs of the *i*-th syllable in the voice input, and there are *k* syllables in total in the voice input. If ${\bf{d}}{\bf{t}}{\bf{w}}({{\boldsymbol{m}}}_{i},{{\boldsymbol{w}}}_{{f}_{i}}) < 200$, ${{N}}_{{f}_{i}}^{{{F}}_{s}}$ is activated; otherwise, the network recognizes ${{\boldsymbol{m}}}_{i}$ as a new feature and creates a new feature neuron to record it: ${N}_{new}^{{F}_{s}}=\{{{\boldsymbol{m}}}_{{i}},1\}$. Then, ${N}_{new}^{{F}_{s}}$ is activated. Finally, the activated feature neurons in the syllable area (assumed to be ${N}_{{{f}}_{{i}}}^{{F}_{s}}$, $1\le i\le k$) transmit their activation signals to the auditory unimodal association area.

Meanwhile, self-organization is conducted among feature neurons using the competitive Hebbian rule (Supplementary Figs. 4–6 show examples of self-organization among feature neurons).

When the visual unimodal association area and auditory unimodal association area receive the activation signals $({N}_{{{f}}_{{b}}}^{{F}_{b}}=1,{N}_{{{f}}_{{b}}}^{{F}_{b}}=1)$ and $({N}_{{{f}}_{1}}^{{F}_{s}}=1,{N}_{{{f}}_{2}}^{{F}_{s}}=1,\ldots ,{N}_{{{f}}_{{k}}}^{{F}_{s}}=1)$, a unimodal-concept incremental learning process is executed.

In the visual channel, if the activation signal $({N}_{{{f}}_{{b}}}^{{F}_{b}}=1,{N}_{{{f}}_{{b}}}^{{F}_{b}}=1)$ satisfies the activation function of a visual concept neuron ${{N}}_{{{f}}_{{v}}}^{{{C}}_{v}}$, which means that the activation function of ${{N}}_{{{f}}_{{v}}}^{{{C}}_{v}}$ is $$f({{N}}_{{{f}}_{{a}}}^{{{F}}_{{a}}},{{N}}_{{{f}}_{{c}}}^{{{F}}_{{c}}})=\{\begin{array}{ll}1, & {{N}}_{{{f}}_{{a}}}^{{{F}}_{{a}}}=1,{{N}}_{{{f}}_{{c}}}^{{{F}}_{{c}}}=1\\ 0, & {\rm{else}}\end{array}$$, then ${{N}}_{{{f}}_{{v}}}^{{{C}}_{v}}$ is activated and the cumulative number of activations of the connection between ${{N}}_{{{f}}_{{v}}}^{{{C}}_{v}}$ and ${N}_{{{f}}_{{b}}}^{{F}_{b}}$, ${{N}}_{{{f}}_{{v}}}^{{{C}}_{v}}$ and ${N}_{{{f}}_{{c}}}^{{F}_{c}}$ is increased by one. If the activation signal $({N}_{{{f}}_{{b}}}^{{F}_{b}}=1,{N}_{{{f}}_{{b}}}^{{F}_{b}}=1)$ does not satisfy the activation function of any visual concept neuron, the network assumes the current input is a new concept and creates a new visual concept neuron ${{N}}_{new}^{{{C}}_{v}}$ to record it. Therefore, an order-independent circuit with ${{N}}_{new}^{{{C}}_{v}}$, ${N}_{{{f}}_{{b}}}^{{F}_{b}}$, and ${N}_{{{f}}_{{b}}}^{{F}_{b}}$ is introduced into the network. Then, ${{N}}_{new}^{{{C}}_{v}}$ is activate. A similar process is executed in the auditory channel. Finally, the activated concept neurons in the visual channel (assumed to be ${{N}}_{{{f}}_{{v}}}^{{{C}}_{v}}$) and auditory channel (assumed to be ${{N}}_{{{f}}_{{a}}}^{{{C}}_{a}}$) transmit their activation signals to the multimodal association area.

When the multimodal association area receives signals ${N}_{{{f}}_{{v}}}^{{C}_{v}}=1$ and ${N}_{{{f}}_{{a}}}^{{C}_{a}}=1$, the network first checks whether ${N}_{{{f}}_{{v}}}^{{C}_{v}}=1$ and ${N}_{{{f}}_{{a}}}^{{C}_{a}}=1$ can activate some association neurons. I assume that the association neurons in set ${N}_{{v}}^{A}$ can be activated by ${N}_{{{f}}_{{v}}}^{{C}_{v}}=1$ and that association neurons in set ${N}_{{a}}^{A}$ can be activated by ${N}_{{{f}}_{{a}}}^{{C}_{a}}=1$. Next, the network activates the auditory concept neurons that connect to the association neurons in ${N}_{{v}}^{A}$ and the visual concept neurons that connect to the association neurons in ${N}_{{a}}^{A}$. I use sets ${N}_{{v}}^{{C}_{a}}$ and ${N}_{{a}}^{{C}_{v}}$ to represent these auditory concept neurons and visual concept neurons, respectively. Obviously, there are four possible combinations between set ${N}_{{v}}^{A}$ and set ${N}_{{a}}^{A}$.

When ${N}_{{v}}^{A}=\varnothing$ and ${N}_{{a}}^{A}\ne \varnothing$, the view of the current input of the object is new to the network, but the voice has been encountered previously and has been used to name some other views. However, the current object should look like the views symbolized by the visual concept neurons in set ${N}_{{a}}^{{C}_{v}}$ according to the current voice input. Now, the network asks the user a question: “I find the current input name ${N}_{{{f}}_{{a}}}^{{C}_{a}}$ has been used to call other views in ${N}_{{a}}^{{C}_{v}}$; can it also represent the current input view?” If the answer from the user is positive, a connection is created between ${N}_{{{f}}_{{v}}}^{{C}_{v}}$ and ${N}_{{{f}}_{{a}}}^{{C}_{a}}$ through each association neuron in set ${N}_{{a}}^{A}$; otherwise, the view ${N}_{{{f}}_{{v}}}^{{C}_{v}}$ is stored as a Type O circuit using a new association neuron.

When ${N}_{{v}}^{A}\ne \varnothing$ and ${N}_{{a}}^{A}=\varnothing$, the current voice input is new to the network, but the view of the current object has been encountered previously. However, the object should be named as symbolized by the auditory concept neurons in set ${N}_{{v}}^{{C}_{a}}$. Therefore, the network asks the user a question: “The object was called ${N}_{{v}}^{{C}_{a}}$ previously. Is it also called ${N}_{{{f}}_{{a}}}^{{C}_{a}}$?” If the answer is positive, a connection is created between ${N}_{{{f}}_{{v}}}^{{C}_{v}}$ and ${N}_{{{f}}_{{a}}}^{{C}_{a}}$ through each association neuron in set ${N}_{{v}}^{A}$; otherwise, the current input name ${N}_{{{f}}_{{a}}}^{{C}_{a}}$ is rejected by the network.

When ${N}_{{v}}^{A}\ne \varnothing$ and ${N}_{{a}}^{A}\ne \varnothing$, both the view and the voice input have been encountered previously. Their coherence should be checked. When ${N}_{{v}}^{A}\cap {N}_{{a}}^{A}\ne \varnothing$, then ${{N}}_{{{f}}_{{v}}}^{{{C}}_{v}}$ and ${{N}}_{{{f}}_{{a}}}^{{{C}}_{a}}$ activate some association neurons in common. The current image/voice pair is consistent with some previous pairs. The cumulative number of activations of the connections between ${{N}}_{{{f}}_{{v}}}^{{{C}}_{v}}$ and ${{N}}_{{{f}}_{{a}}}^{{{C}}_{a}}$ through the activated association neurons in common are increased by one to strengthen the association. When ${N}_{{v}}^{A}\cap {N}_{{a}}^{A}=\varnothing$, it means the current combination of ${{N}}_{{{f}}_{{v}}}^{{{C}}_{v}}$ and ${{N}}_{{{f}}_{{a}}}^{{{C}}_{a}}$ is inconsistent with previous pairs. Therefore, the network asks the user a question: “The current input pair is inconsistent with previous pairs, is this pair an expected combination?” If the answer is positive, the connections between ${N}_{{{f}}_{{v}}}^{{C}_{v}}$ and ${N}_{{{f}}_{{a}}}^{{C}_{a}}$ through the activated association neurons in common are created; otherwise, the current input pair is rejected by the network.

When ${N}_{{v}}^{A}=\varnothing$ and ${N}_{{a}}^{A}=\varnothing$, the current combination of ${{N}}_{{{f}}_{{v}}}^{{{C}}_{v}}$ and ${{N}}_{{{f}}_{{a}}}^{{{C}}_{a}}$ has not been encountered previously. A new association neuron is created to associate ${{N}}_{{{f}}_{{v}}}^{{{C}}_{v}}$ and ${{N}}_{{{f}}_{{a}}}^{{{C}}_{a}}$ with a 1-to-1 circuit.

At this point, the entire learning process for the visual and auditory input pair is complete, and the network continues with the next input pair.

### Dimensionality increasing process of the feature neurons

When novel receptors are added to an existing perceptual channel, feature neurons handle the signals from the novel receptors. As illustrated in Fig. 3c, new synapses will grow to connect the new sensory receptors, which means that the spectrum range of the feature neuron is broadened by the new receptors. I call it a dimensionality increasing process.

I denote ${{\boldsymbol{x}}}_{{\bf{I}}}=({x}_{1},{x}_{2},\ldots ,{x}_{n})\in {{\bf{R}}}^{n}$ as the data received from the originally existing $n$ sensory neurons, ${{\boldsymbol{x}}}_{{\bf{II}}}=({x}_{n+1},{x}_{n+2},\ldots ,{x}_{n+m})\in {{\bf{R}}}^{m}$ as the data received from the $m$ new sensory neurons, ${{S}}_{{\rm{I}}}={{\bf{R}}}^{n}$ as the original sensory space, and ${{S}}_{{\rm{II}}}={{\bf{R}}}^{n+m}$ as the new sensory receptor space. During the dimensionality increasing process, two types of feature neurons can exist: type one consists of neurons that already completed the dimensionality increasing process, and type two is the neurons that have not completed the dimensionality increasing process. I use the sets ${N}_{{\rm{I}}}^{{F}_{\alpha }}$ and ${N}_{{\rm{II}}}^{{F}_{\alpha }}$ to represent the type one and type two feature neurons, respectively, and use ${N}^{{F}_{\alpha }}={N}_{{\rm{I}}}^{{F}_{\alpha }}\cup {N}_{{\rm{II}}}^{{F}_{\alpha }}$ to store the feature neurons in area *α*.

When an input sample ${\boldsymbol{x}}=({{\boldsymbol{x}}}_{{\bf{I}}},{{\boldsymbol{x}}}_{{\bf{II}}})$ arrives, feature extraction is conducted using ${{\boldsymbol{x}}}_{{\bf{I}}}$ and ${{\boldsymbol{x}}}_{{\bf{II}}}$ first, ${{\boldsymbol{y}}}_{{\rm{I}}}=g({{\boldsymbol{x}}}_{{\rm{I}}}),{{\boldsymbol{y}}}_{{\rm{II}}}=g({{\boldsymbol{x}}}_{{\rm{II}}})$, where *g*(·) is the feature extraction function, and ${\boldsymbol{y}}=({{\boldsymbol{y}}}_{{\rm{I}}},{{\boldsymbol{y}}}_{{\rm{II}}})$ is the total number of features in the current input sample ***x***. Next, among the neurons in set ${N}^{{F}_{\alpha }}$, a winner neuron is found through competition in the original sensory space *S*_I_, such that ${N}_{a}^{{F}_{\alpha }}=\mathop{{\rm{\arg }}\,{\rm{\min }}}\limits_{{N}_{j}^{{F}_{\alpha }}\in {N}^{{F}_{\alpha }}}\,{\boldsymbol{D}}({{\boldsymbol{y}}}_{{\rm{I}}},{\hat{{\boldsymbol{w}}}}_{j})$, where ${\hat{{\boldsymbol{w}}}}_{j}=({w}_{1},{w}_{2},\ldots ,{w}_{n})$ represents the weight vector of ${N}_{j}^{{F}_{\alpha }}$ in space *S*_I_, and function ***D***(·,·) measures the similarity between the two variables. Among the neurons in set ${N}_{{\rm{II}}}^{{F}_{\alpha }}$, a winner neuron is found in the evolved sensory space *S*_II_, such that ${N}_{b}^{{F}_{\alpha }}=\mathop{{\rm{\arg }}\,{\rm{\min }}}\limits_{{N}_{j}^{{F}_{\alpha }}\in {N}_{{\rm{II}}}^{{F}_{\alpha }}}\,D({\boldsymbol{y}},{{\boldsymbol{w}}}_{j})$, where ${\hat{{\boldsymbol{w}}}}_{j}=({w}_{1},{w}_{2},\ldots ,{w}_{n+m})$ represents the weight vector of ${N}_{j}^{{F}_{\alpha }}$ in space *S*_II_. Then, I check whether ***y***_I_ and ***y*** can activate the feature neurons ${N}_{a}^{{F}_{\alpha }}$ and ${N}_{b}^{{F}_{\alpha }}$, respectively, which requires calculating equations ${f}_{{N}_{a}^{{F}_{\alpha }}}({{\boldsymbol{y}}}_{{\rm{I}}})$ and ${f}_{{N}_{b}^{{F}_{\alpha }}}({\boldsymbol{y}})$, where ${f}_{{N}_{a}^{{F}_{\alpha }}}$(·) and ${f}_{{N}_{b}^{{F}_{\alpha }}}$(·) are the activation functions of ${N}_{a}^{{F}_{\alpha }}$ and ${N}_{b}^{{F}_{\alpha }}$, respectively.

If ${f}_{{N}_{a}^{{F}_{\alpha }}}({{\boldsymbol{y}}}_{{\rm{I}}})=1$ and ${f}_{{N}_{b}^{{F}_{\alpha }}}({\boldsymbol{y}})=0$, then ***y***_I_ activates ${N}_{a}^{{F}_{\alpha }}$ but ***y*** does not activate ${N}_{b}^{{F}_{\alpha }}$. This implies that a familiar feature **y**_I_ is accompanied by a novel feature in the unity form ***y*** that is not recognized by the network. As a result, the dimensionality increasing process should be applied to ${N}_{a}^{{F}_{\alpha }}$:

4 $$\begin{array}{l}{\sigma }_{{a}}={\sigma }_{{a}}+1,\,{{\boldsymbol{w}}}_{{a}}={{\boldsymbol{w}}}_{{a}}+\frac{1}{{\sigma }_{{a}}}({{\boldsymbol{y}}}_{{\rm{I}}}-{{\boldsymbol{w}}}_{{a}})\\ {{\boldsymbol{w}}}_{{a}}=({{\boldsymbol{w}}}_{{a}},{{\boldsymbol{y}}}_{{\bf{II}}})\end{array}$$

where ${\sigma }_{{a}}$ represents the cumulative number of times that ${N}_{a}^{{F}_{\alpha }}$ has been activated and ${{\boldsymbol{w}}}_{{a}}$ is the weight vector of ${N}_{a}^{{F}_{\alpha }}$. It is worth noting that Eq. (4) increases the number of synapses for a neuron. The weights of the new added synapses are set to ***y***_II_ which causes ${N}_{a}^{{F}_{\alpha }}$ to connect to the novel feature ***y***_II_. Consequently, the response range of ${N}_{a}^{{F}_{\alpha }}$ is broadened. Equation (4) denotes the dimensionality increasing process for a feature neuron.

When ${f}_{{N}_{a}^{{F}_{\alpha }}}({{\boldsymbol{y}}}_{{\rm{I}}})=1$ and ${f}_{{N}_{b}^{{F}_{\alpha }}}({\boldsymbol{y}})=1$, then ***y***_I_ activates ${N}_{a}^{{F}_{\alpha }}$ and ${\boldsymbol{y}}$ activates ${N}_{b}^{{F}_{\alpha }}$. Therefore, the input feature ${\boldsymbol{y}}$ is recognized by the network. In this situation, ${N}_{b}^{{F}_{\alpha }}$ inhibits ${N}_{a}^{{F}_{\alpha }}$, and ${N}_{b}^{{F}_{\alpha }}$ is updated as follows:

$${\sigma }_{{b}}={\sigma }_{{b}}+1,\,{{\boldsymbol{w}}}_{{b}}={{\boldsymbol{w}}}_{{b}}+\frac{1}{{\sigma }_{{b}}}({\boldsymbol{y}}-{{\boldsymbol{w}}}_{{b}})$$

When ${f}_{{N}_{a}^{{F}_{\alpha }}}({{\boldsymbol{y}}}_{{\rm{I}}})=0$ and ${f}_{{N}_{b}^{{F}_{\alpha }}}({\boldsymbol{y}})=1$, then ***y***_I_ does not activate ${N}_{a}^{{F}_{\alpha }}$ but ${\boldsymbol{y}}$ activates ${N}_{b}^{{F}_{\alpha }}$. The input feature ***y*** is recognized by the network, and ${N}_{b}^{{F}_{\alpha }}$ is updated using the above equations.

When ${f}_{{N}_{a}^{{F}_{\alpha }}}({{\boldsymbol{y}}}_{{\rm{I}}})=0$ and ${f}_{{N}_{b}^{{F}_{\alpha }}}({\boldsymbol{y}})=0$, it means ***y***_I_ does not activate ${N}_{a}^{{F}_{\alpha }}$ and ${\boldsymbol{y}}$ does not activate ${N}_{b}^{{F}_{\alpha }}$. A new feature neuron ${N}_{new}^{{F}_{\alpha }}=\{{\boldsymbol{y}},1\}$ is created to record the input feature ${\boldsymbol{y}}$. Then, ${N}_{new}^{{F}_{\alpha }}$ is activated.

Finally, the activation signal generated by the activated feature neuron is transmitted to the unimodal association areas.

### Response modal expansion of the association neurons

When a new perceptual channel ${\bf{X}}$ is added to the network, the association neurons handle the signals transmitted from ${\bf{X}}$. As illustrated in Fig. 3e, new synapses will grow to connect the concept neurons in channel ${\bf{X}}$, which implies that the response modal of the association neuron is extended.

I denote ${{\boldsymbol{x}}}^{{\bf{I}}}=({x}_{1}^{{\bf{I}}},{x}_{2}^{{\bf{I}}},\ldots ,{x}_{n}^{{\bf{I}}})\in {{\bf{R}}}^{n}$ as the data received from the pre-existing perceptual channel ${\bf{L}}$ and ${{\boldsymbol{x}}}^{{\bf{II}}}=({x}_{1}^{{\bf{II}}},{x}_{2}^{{\bf{II}}},\ldots ,{x}_{m}^{{\bf{II}}})\in {{\bf{R}}}^{m}$ as the data received from the new channel ${\bf{X}}$.

When an input sample pair $({{\boldsymbol{x}}}^{{\bf{I}}},{{\boldsymbol{x}}}^{{\bf{II}}})$ arrives, ${{\boldsymbol{x}}}^{{\bf{I}}}$ and ${{\boldsymbol{x}}}^{{\bf{II}}}$ activate the concept neurons (the same as in Algorithm 1, steps 2 and 3; therefore, I omit them here). Assume that the concept neurons ${N}_{{a}}^{{C}_{L}}$ and ${N}_{{b}}^{{C}_{X}}$ are activated in channels ${\bf{L}}$ and ${\bf{X}}$, respectively. Then, ${N}_{{a}}^{{C}_{L}}$ and ${N}_{{b}}^{{C}_{X}}$ transmit their activated signals to the multimodal association areas to activate the association neurons. Assume the association neurons in set ${N}_{{L}}^{A}$ are activated by ${N}_{{a}}^{{C}_{L}}$ and the association neurons in set ${N}_{{X}}^{A}$ are activated by ${N}_{{b}}^{{C}_{X}}$. Obviously, four combinations exist between set ${N}_{{L}}^{A}$ and set ${N}_{{X}}^{A}$.

When ${N}_{{X}}^{A}=\varnothing$ and ${N}_{{L}}^{A}\ne \varnothing$, then ${{\boldsymbol{x}}}^{{\bf{I}}}$ activates some association neurons but ${{\boldsymbol{x}}}^{{\bf{II}}}$ does not. That is, ${{\boldsymbol{x}}}^{{\bf{I}}}$ has been encountered previously, but ${{\boldsymbol{x}}}^{{\bf{II}}}$ is new to the network. The network obtains a new modality feature for the current object through channel ${\bf{X}}$. The association neurons in ${N}_{{L}}^{A}$ should associate ${N}_{{b}}^{{C}_{X}}$ with ${N}_{{a}}^{{C}_{L}}$. Thus, a new connection is created between ${N}_{{b}}^{{C}_{X}}$ and ${N}_{{a}}^{{C}_{L}}$ through each association neuron in set ${N}_{{L}}^{A}$:

5 $${c}_{(m,i,n)}=\{{{N}}_{{a}}^{{{C}}_{L}},{{N}}_{{i}}^{{A}},{{N}}_{{b}}^{{{C}}_{X}},1\},\,{\rm{where}}\,{{N}}_{{i}}^{{A}}\in {{N}}_{{L}}^{{A}}$$

It is worth noting that Eq. (5) connects the concept neurons in channel ${\bf{X}}$ to the association neuron which was not previously able to respond to channel ${\bf{X}}$. Thus, this process realizes the response-modal expansion process of the association neuron. Subsequently, the network can respond to channel ${\bf{X}}$.

When ${N}_{{X}}^{A}\ne \varnothing$ and ${N}_{{L}}^{A}\ne \varnothing$, both ${{\boldsymbol{x}}}^{{\bf{I}}}$ and ${{\boldsymbol{x}}}^{{\bf{II}}}$ activate some association neurons, and ${{\boldsymbol{x}}}^{{\bf{I}}}$ and ${{\boldsymbol{x}}}^{{\bf{II}}}$ have been encountered previously. When ${N}_{{X}}^{A}\cap {N}_{{L}}^{A}\ne \varnothing$, ${{\boldsymbol{x}}}^{{\bf{I}}}$ and ${{\boldsymbol{x}}}^{{\bf{II}}}$ activate some association neurons in common. The current input pair ${{\boldsymbol{x}}}^{{\bf{I}}}$ and ${{\boldsymbol{x}}}^{{\bf{II}}}$ is an encountered pair. Consequently, the cumulative number of activations of the connections between ${N}_{{a}}^{{C}_{L}}$ and ${N}_{{b}}^{{C}_{X}}$ through the activated association neurons they have in common are increased by one to strengthen the association:

$${\rho }_{(a,i,b)}={\rho }_{(a,i,b)}+1,\,where\,{{N}}_{{i}}^{{A}}\in {{N}}_{{L}}^{{A}}\cap {{N}}_{{X}}^{{A}}$$

when ${N}_{{v}}^{A}\cap {N}_{{a}}^{A}=\varnothing$, the current input pair ${{\boldsymbol{x}}}^{{\bf{I}}}$ and ${{\boldsymbol{x}}}^{{\bf{II}}}$ is not an encountered pair. New connections are created between ${N}_{{a}}^{{C}_{L}}$ and ${N}_{{b}}^{{C}_{X}}$ to bind them,

$${c}_{(m,i,n)}=\{{{N}}_{{a}}^{{{C}}_{L}},{{N}}_{{i}}^{{A}},{{N}}_{{b}}^{{{C}}_{X}},1\},\,where\,{{N}}_{{i}}^{{A}}\in {{N}}_{{L}}^{{A}}\cup {{N}}_{{X}}^{{A}}$$

when ${N}_{{X}}^{A}\ne \varnothing$ and ${N}_{{L}}^{A}=\varnothing$, then ${{\boldsymbol{x}}}^{{\bf{II}}}$ activates some association neurons but ${{\boldsymbol{x}}}^{{\bf{I}}}$ does not. The association neurons in ${N}_{{X}}^{A}$ will associate ${N}_{{a}}^{{C}_{L}}$ with ${N}_{{b}}^{{C}_{X}}$. New connections are created between ${N}_{{a}}^{{C}_{L}}$ and ${N}_{{b}}^{{C}_{X}}$ through the association neurons in set ${N}_{{X}}^{A}$:

$${c}_{(m,i,n)}=\{{{N}}_{{a}}^{{{C}}_{L}},{{N}}_{{i}}^{{A}},{{N}}_{{b}}^{{{C}}_{X}},1\},\,where\,{{N}}_{{i}}^{{A}}\in {{N}}_{{X}}^{{A}}$$

when ${N}_{{X}}^{A}=\varnothing$ and ${N}_{{L}}^{A}=\varnothing$, neither ${{\boldsymbol{x}}}^{{\bf{I}}}$ nor ${{\boldsymbol{x}}}^{{\bf{II}}}$ activates any association neurons, and the concepts ${N}_{{b}}^{{C}_{X}}$ and ${N}_{{a}}^{{C}_{L}}$ are new to the network. A new association neuron is created to connect ${N}_{{b}}^{{C}_{X}}$ and ${N}_{{a}}^{{C}_{L}}$ with a 1-to-1 circuit.

At this point, the learning for the current input pair $({{\boldsymbol{x}}}^{{\bf{I}}},{{\boldsymbol{x}}}^{{\bf{II}}})$ is complete, and the network continues with the next pair.

### Modality embedding

To embed a new sensory modality in some particular neurons in an expected channel ${\bf{T}}$, a guidance signal is needed to guide the new sensory input to a target neuron in channel ${\bf{T}}$. Thus, the input format is $({{\boldsymbol{x}}}_{t},{{\boldsymbol{x}}}_{{g}})$, where ${{\boldsymbol{x}}}_{t}$ is the sample to be embedded and ${{\boldsymbol{x}}}_{{g}}$ is the guidance signal that will be fed into the guidance channel ${\bf{G}}$.

When the network receives $({{\boldsymbol{x}}}_{t},{{\boldsymbol{x}}}_{{g}})$, it uses the guidance signal ${{\boldsymbol{x}}}_{{g}}$ to find the target neuron in channel ${\bf{T}}$ that will absorb ${{\boldsymbol{x}}}_{t}$. Through channel ${\bf{G}}$, some association neurons are activated by ${{\boldsymbol{x}}}_{{g}}$. Then, these association neurons activate the concept neurons to which they are connected in channel ${\bf{T}}$, and subsequently, the concept neurons activate their feature neurons. The activated neurons in both channel ${\bf{G}}$ and channel ${\bf{T}}$ form a neural circuit that will be reused for the novel modality.

Assume that concept neuron ${N}_{{a}}^{{C}_{T}}$ and feature neurons ${N}_{1}^{{F}_{T}}$, ${N}_{2}^{{F}_{T}}$, …, ${N}_{{m}}^{{F}_{T}}$ are activated in channel ${\bf{T}}$. In the following, I choose feature neuron ${N}_{{f}}^{{F}_{T}}$ as the target neuron to be embedded with ${{\boldsymbol{x}}}_{t}$. The embedding can be divided into two situations: (1) ${N}_{{f}}^{{F}_{T}}$ has not previously been embedded with the novel modality and (2) ${N}_{{f}}^{{F}_{T}}$ has already been embedded with the novel modality.

For situation one, neuron ${N}_{{f}}^{{F}_{T}}$ creates new synapses to connect ${{\boldsymbol{x}}}_{t}$. This means that neuron ${N}_{{f}}^{{F}_{T}}$ now has two sets of synapses: one is the original signal pathway, and the other is used to transmit the exogenous signals. The weights of these expanded synapses are initialized to ${{\boldsymbol{x}}}_{t}$,

6 $${{\boldsymbol{w}}}_{{f}}=\{({{\boldsymbol{w}}}_{{f}}),\,({{\boldsymbol{x}}}_{{t}})\}$$

In the equation, I use the parentheses to separate the different sets of synaptic weights. Thus, the novel modality is embedded in the target neuron.

For situation two, the weights of the expanded synapses are updated with sample ${{\boldsymbol{x}}}_{t}$ as follows:

7 $${\sigma }_{{f}}^{{\rm{II}}}={\sigma }_{{f}}^{{\rm{II}}}+1,\,{{\boldsymbol{w}}}_{{f}}^{{\rm{II}}}={{\boldsymbol{w}}}_{{f}}^{{\rm{II}}}+\frac{1}{{\sigma }_{{f}}^{{\rm{II}}}}({{\boldsymbol{x}}}_{t}-{{\boldsymbol{w}}}_{{f}}^{{\rm{II}}})$$

where ${{\boldsymbol{w}}}_{{f}}^{{\rm{II}}}$ represents the weights of the expanded synapses, and ${\sigma }_{{f}}^{{\rm{II}}}$ represents the cumulative number of times these synapses have been activated.

At this point, the embedding process for the current input sample is finished, and the network continues with the next input sample.

## Supplementary information

A Mathematical Theory of Cortex-Receptor Artificial Extension.

## Data Availability

Matlab code and data are available at https://github.com/cloudlee711/CREAT.
