# Supplementary material for: A Mathematical Theory of Cortex-Receptor Artificial Extension
Source: Sci Rep. 2020 Jan 21;10:765. doi: 10.1038/s41598-020-57591-w (PMC6972759; doi:10.1038/s41598-020-57591-w)
Supplement: Supplementary file 1 — A Mathematical Theory of Cortex-Receptor Artificial Extension. [file 41598_2020_57591_MOESM1_ESM.docx]

Supplementary Materials for

A Mathematical Theory of Cortex-Receptor Artificial Extension

You-Lu Xing

Correspondence to: [youluxing@sina.c](mailto:xxxxx@xxxx.xxx)om

**This file includes:**

Section 1. Details of the learning data.

Supplementary Fig. 1. Objects used in the experiment.

Supplementary Fig. 2. Examples of the experimental results.

Supplementary Fig. 3. Examples of the modality embedding result.

Supplementary Fig. 4. Self-organizing result of the color feature neurons.

Supplementary Fig. 5. Self-organizing result of the shape feature neurons.

Supplementary Fig. 6. Examples of the self-organizing result of the syllable feature neurons.

1. **Details of the learning data**

As shown in Fig. S1, twenty objects are used in the experiments. Because I did not have real inaudible sound data and taste data, I designed an artificial data set. For the inaudible sound data, I generated 35 types of sine waves at every 3 kHz with an amplitude of 2; their frequency ranged from 20 kHz to 122 kHz. The sampling frequency was 1 mHz. Random noise was added to the sine waves. I denote the 35 types of sine waves as $u_{i}$, $1\leq i\leq35$. Artificial words were generated using these high frequency waves and Chinese syllables; for example, I used “$u_{1}\to u_{2}$” and “$u_{1}\to s_{2}$” as the name for an apple, where $s_{2}$is a Chinese syllable. I designed different combinations of one to three sine waves $u_{i}$ and Chinese syllables $s_{j}$as names for different objects. The adjacent sine waves and Chinese syllables were linked by a background wave $bg$, consisting of random noise. This means that the artificial words have the forms $[u_{i}]$, $[u_{i},bg,u_{j},...]$, $[u_{i},bg,s_{j},...]$, and $[s_{i},bg,u_{j},...]$ and contain one to three syllables. For the taste feature data, I used a 6-dimensional vector, (sweet, sour, salt, bitter, umami, hot). The value of each attribute was within the range of [0, 1]. For example, I designed the taste of an apple as follows: the sweet value was uniformly distributed in the range [0.5, 0.6]; the sour value was uniformly distributed in the range [0, 0.1]; and the other attributes were set to 0.

| **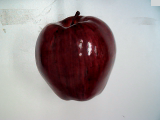** | **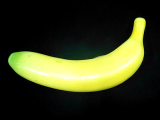** | **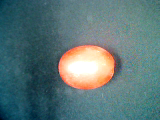** | **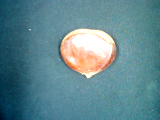** | **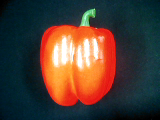** |
| --- | --- | --- | --- | --- |
| **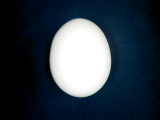** | **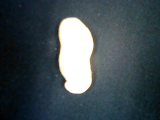** | **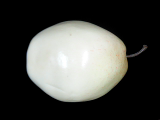** | **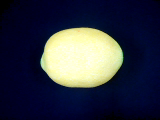** | **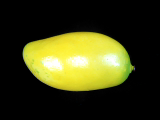** |
| **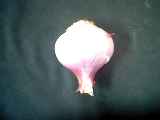** | **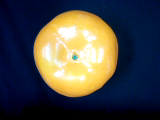** | **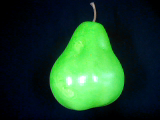** | **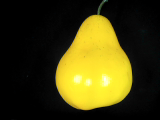** | **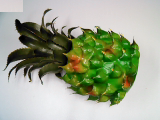** |
| **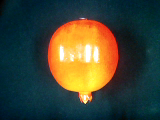** | **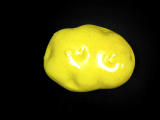** | **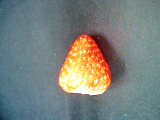** | **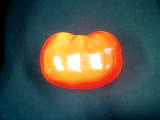** | **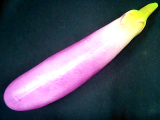** |

**Supplementary Fig. 1: Objects used in the experiment.**


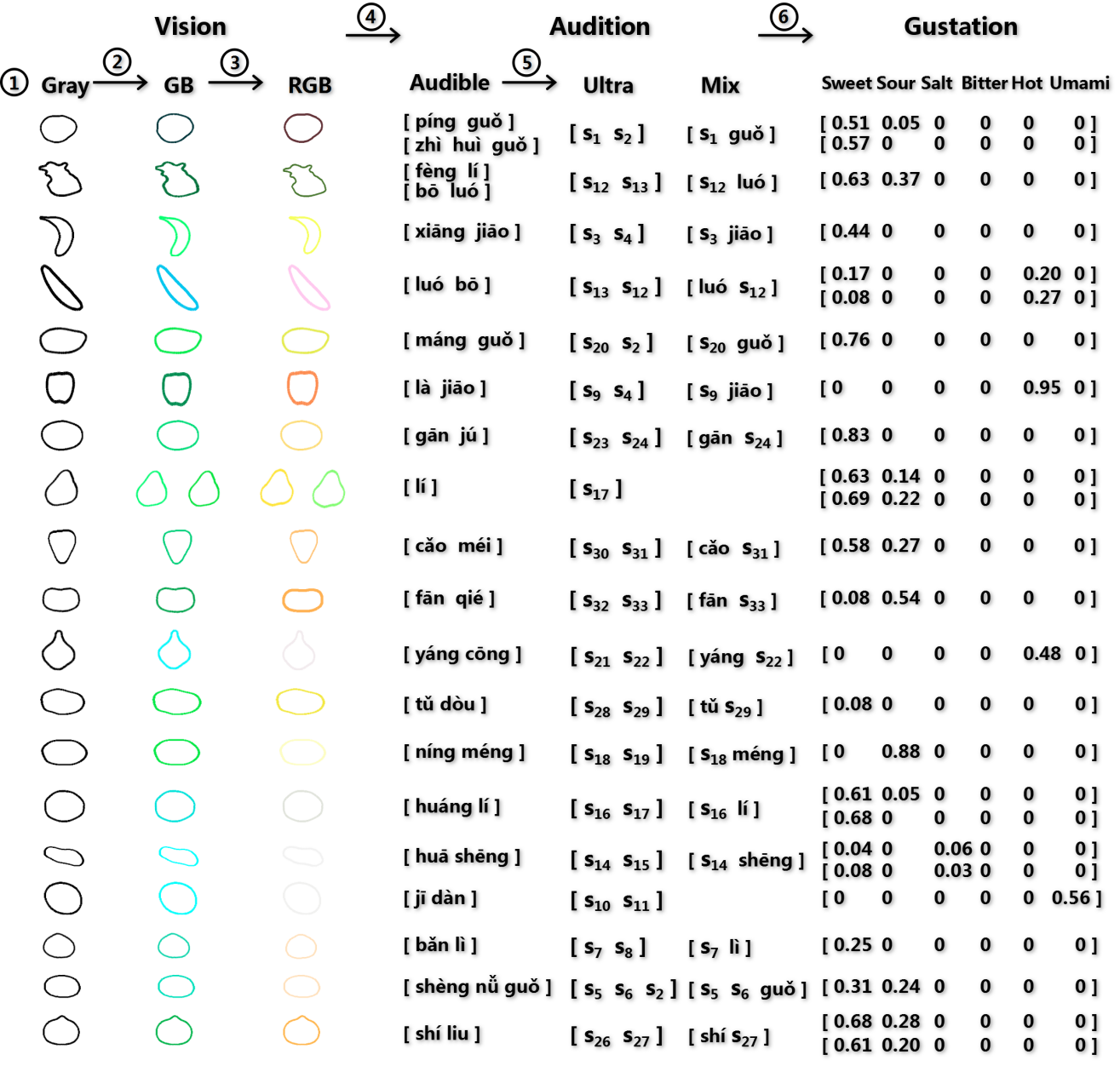


Supplementary Fig. 2: Examples of the experimental results. Each column represents the concepts learned (or updated from existing concepts) by the CRAET network. Each row shows concepts associated by the network, and the network usually learns more than one association per object.


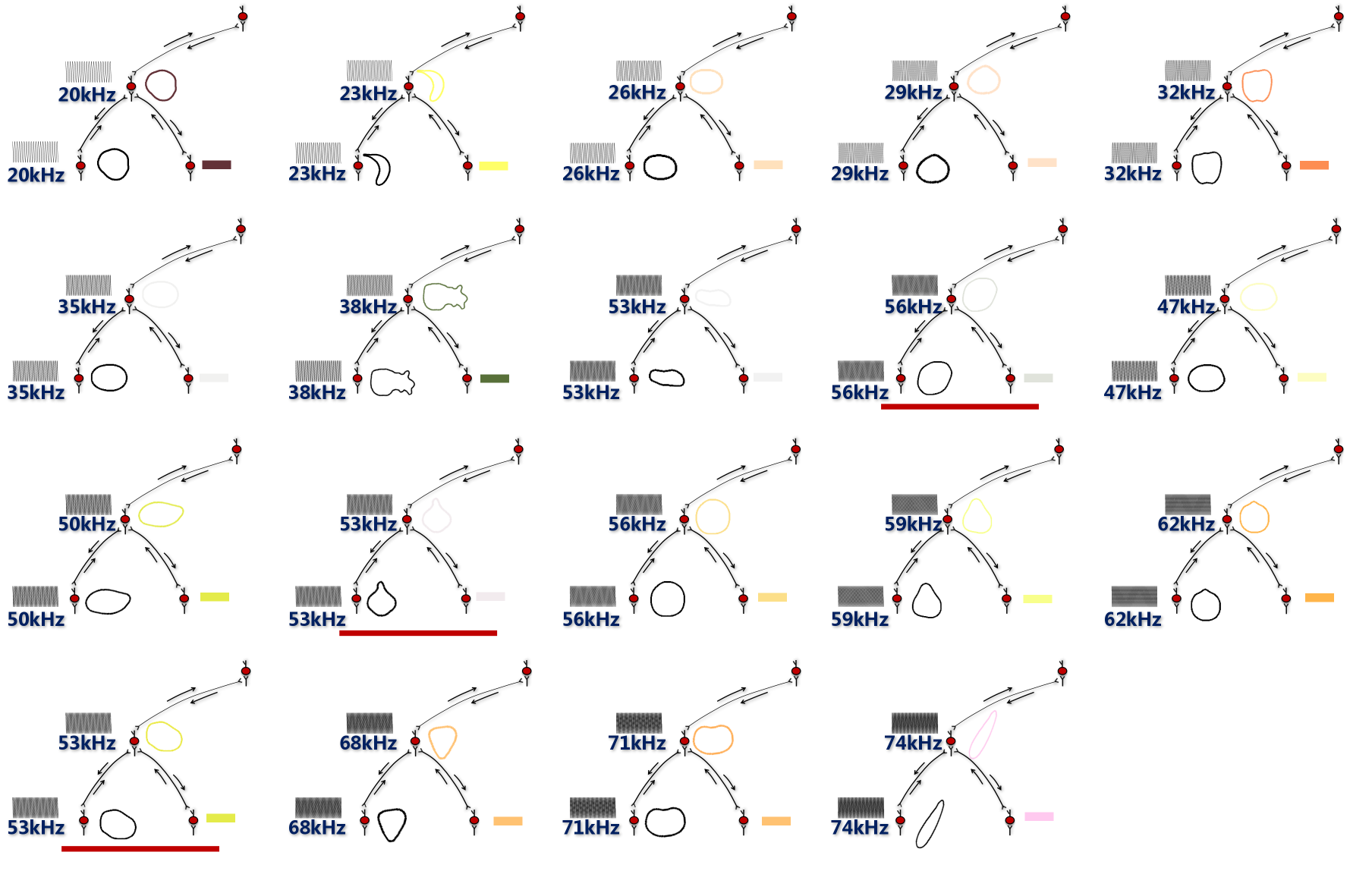


Supplementary Fig. 3: Examples of the modality embedding result. The icons next to the neurons represent the objects to which the neurons maximally respond. The red underlined results respond to unexpected ultrasound frequencies. These errors are due to the association neurons mistakenly binding views of different objects.

**
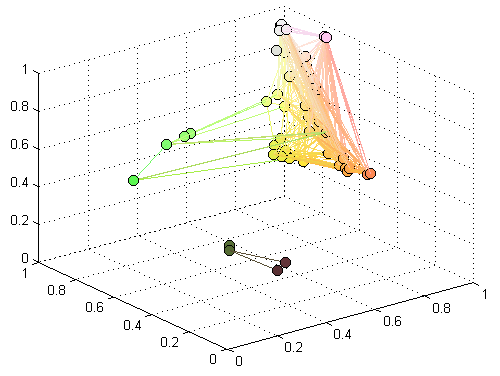
**

Supplementary Fig. 4. Self-organizing result of the color feature neurons. The color features are organized by Euclidean distance. Similar colors are connected.

**
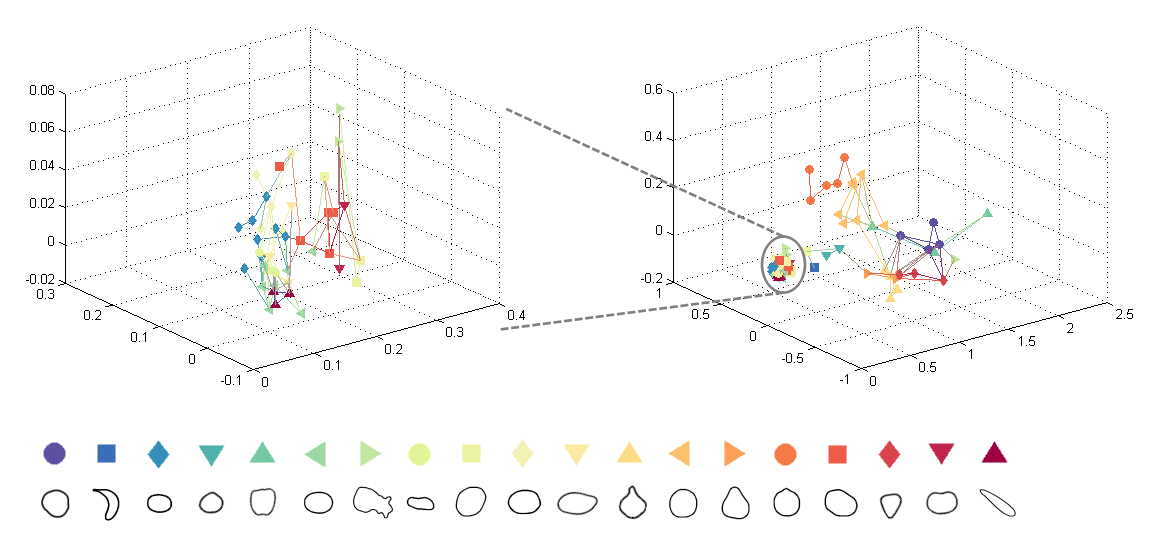
**

Supplementary Fig. 5: Self-organizing result of the shape feature neurons. The shape features are organized by Euclidean distance, and similar shapes are connected.


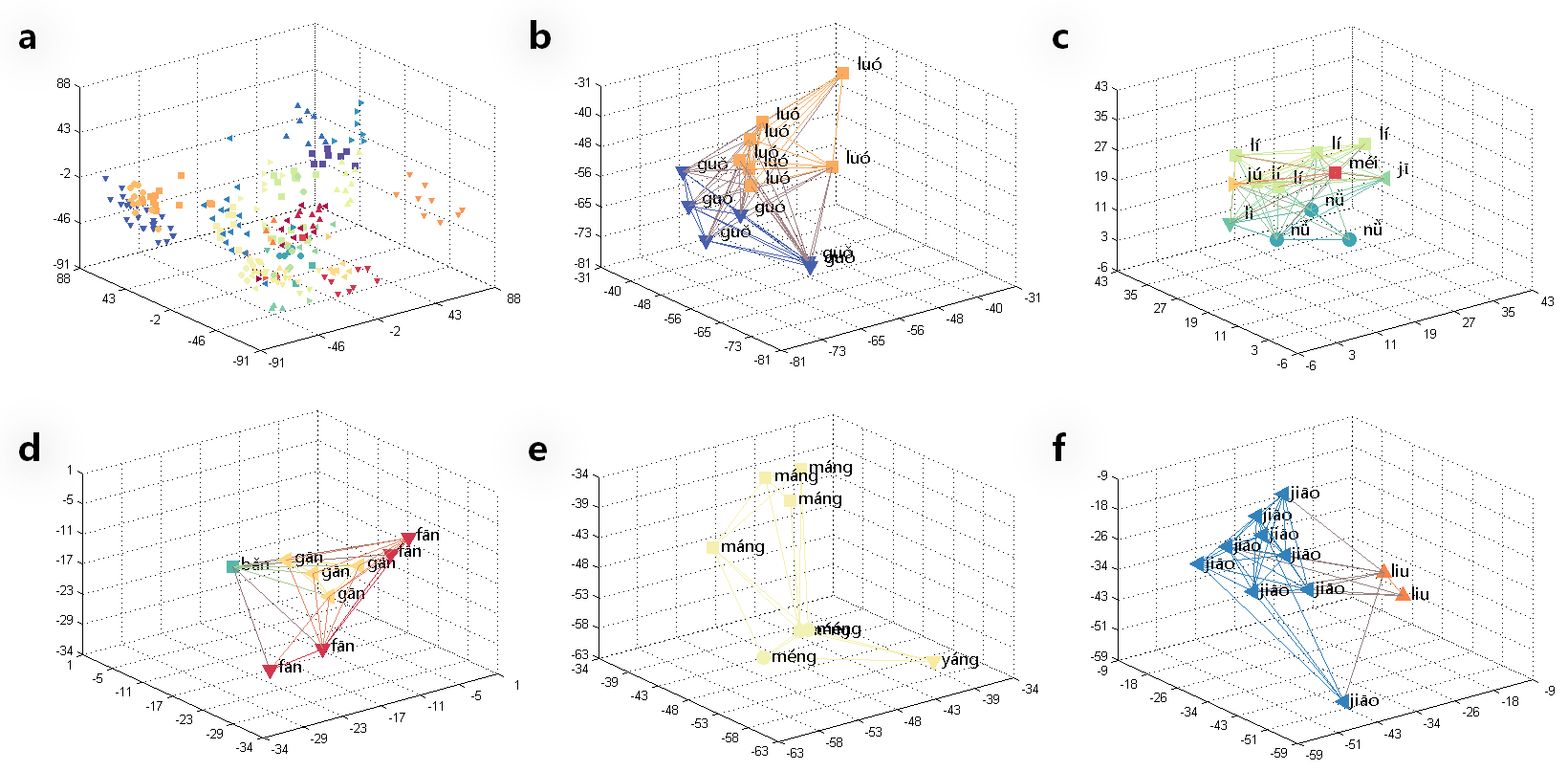


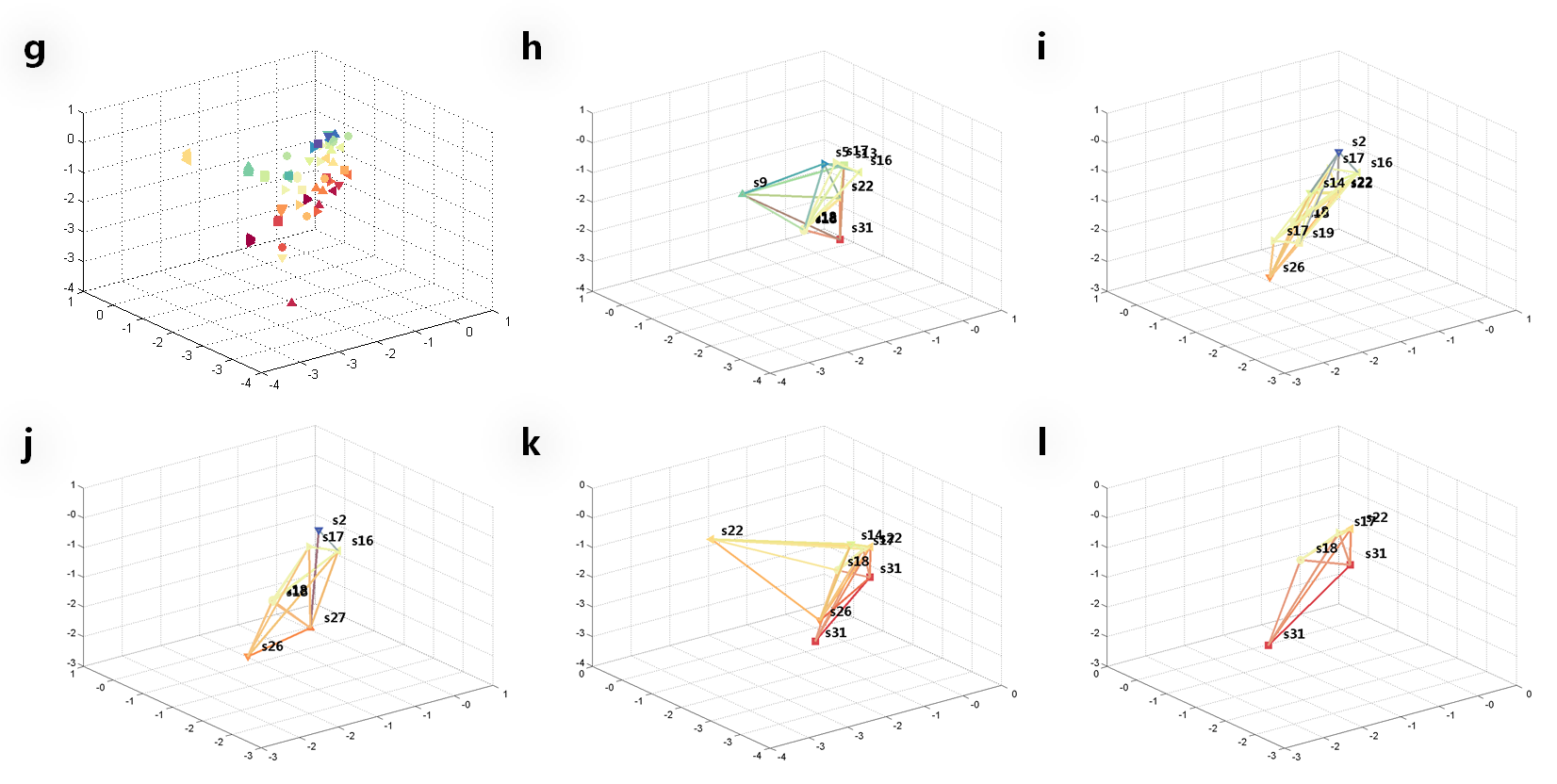


Supplementary Fig. 6: Examples of the self-organizing result of the syllable feature neurons. (a) the learned Chinese syllables. Each point represents a Chinese syllable visualized by principal component analysis. (b-e) Self-organizing result of five subsets of Chinese syllable neurons. The syllable features are organized by the Dynamic Time Warping distance, and similar syllables are connected. (g) learned ultrasonic syllables. Each point represents an ultrasonic syllable, which is visualized by principal component analysis. (h-l) Self-organizing result of five subsets of the ultrasonic syllable neurons. The syllable features are organized by the Dynamic Time Warping distance.
